# Long-term outcome of a moderately hypofractionated, intensity-modulated radiotherapy approach using an endorectal balloon for patients with localized prostate cancer

**DOI:** 10.1186/s40880-018-0281-4

**Published:** 2018-04-17

**Authors:** Bin S. Teh, Gary D. Lewis, Weiyuan Mai, Ramiro Pino, Hiromichi Ishiyama, Edward Brian Butler

**Affiliations:** 1 Department of Radiation Oncology, Houston Methodist Hospital, Cancer Center, and Research Institute, Weill Cornell Medical College, 6565 Fannin, Ste#DB1-077, Houston, TX 77030 USA; 2 Department of Radiation Oncology, The University of Texas Medical Branch at Galveston, Galveston, TX 77555 USA; 3 Department of Radiation Oncology, Baylor College of Medicine, Houston, TX 77030 USA; 4 Department of Radiology and Radiation Oncology, Kitasato University School of Medicine, Sagamihara, 252-0374 Japan

**Keywords:** Prostate cancer, Intensity-modulated radiotherapy, Moderate hypofractionation

## Abstract

**Background:**

Technical advances in radiotherapy delivery have simultaneously enabled dose escalation and enhanced bladder and rectal sparing. However, the optimal radiation fractionation regimen for localized prostate cancer is unclear. Laboratory and clinical evidence suggest that hypofractionation may improve the therapeutic ratio of radiotherapy. We report our institutional outcomes using moderately hypofractionated, intensity-modulated radiotherapy (IMRT), and an endorectal balloon, with emphasis on long-term biochemical control and treatment-related adverse events in patients with localized prostate cancer.

**Methods:**

Between January 1997 and April 2004, 596 patients with cT1–T3 prostate cancer underwent IMRT using a moderate hypofractionation regimen (76.70 Gy at 2.19 Gy/fraction) with an endorectal balloon. Using D’Amico classification, 226 (37.9%), 264 (44.3%), and 106 (17.8%) patients had low-, intermediate-, or high-risk disease, respectively. The majority of intermediate- and high-risk patients received androgen deprivation therapy. Biochemical relapse-free survival (bRFS) was evaluated using 2005 Phoenix criteria and estimated using the Kaplan–Meier method.

**Results:**

The median follow-up was 62 months. Overall 5- and 10-year bRFS rates were 92.7% and 87.7%. For low-, intermediate-, and high-risk patients, the 5-year bRFS rates were 96.9%, 93.3%, and 82.0%, respectively; the 10-year bRFS rates were 91.4%, 89.3%, and 76.2%, respectively. Prostate-specific antigen, Gleason score, and T stage were significant predictors of bRFS (all *P* < 0.01). The 5-year rates of severe (≥ Grade 3) adverse events were very low: 1.2% for gastrointestinal events and 1.1% for genitourinary events.

**Conclusions:**

Long-term outcomes after moderately hypofractionated IMRT are encouraging. Moderate hypofractionation represents a safe, efficacious, alternative regimen in the treatment of localized prostate cancer.

## Background

Dose-escalated radiotherapy is an established treatment for localized prostate cancer. Technical advances in radiation delivery, including three-dimensional conformal radiotherapy (3D-CRT) and, more recently, intensity-modulated radiotherapy (IMRT) have simultaneously enabled dose escalation and enhanced bladder and rectal sparing. These developments have led to demonstrable gains in therapeutic ratio through improved disease control rates and concomitant reductions in acute and chronic adverse events [1, 2].

Evidence from multiple retrospective and prospective series of patients with localized prostate cancer confirmed the theoretical benefits of dose escalation. A mature phase III trial from M.D. Anderson Cancer Center revealed decreases in biochemical and clinical disease progression (including distant metastases) rates for patients treated with an isocenter dose of 78 Gy compared with 70 Gy [3]. Similarly, a randomized trial by Zietman et al. [4] demonstrated benefits from proton boost escalation of 79.2 vs. 70.2 Gy, and a Dutch randomized trial revealed improved freedom from failure for patients receiving 78 vs. 68 Gy [5]. Prospective dose escalation data from Memorial Sloan Kettering Cancer Center suggested similar clinical benefits, including reduced distant failure independent of short-term androgen deprivation therapy (ADT) [6].

Collectively, the above studies reflect progressive gains using conventional fraction sizes of 1.8–2.0 Gy and are validated by updated National Comprehensive Cancer Network (NCCN) guidelines [7]. However, the optimal radiation fractionation regimen for localized prostate cancer is unclear as a result of mounting laboratory and clinical evidence suggesting that the alpha/beta (α/β) ratio for most prostate cancers lies between 1 and 3 Gy [8–10]. This hypothesis provides a scientific rationale for hypofractionation [8–11] and, if verified, would have significant therapeutic implications including improved patient convenience and cost savings by virtue of reduced number of fractions. Data from a handful of prospective trials [12–15] are consistent with the hypothesis. However, significant heterogeneity exists among reported hypofractionation regimens [12–15].

Herein, we report our institutional outcomes using moderately hypofractionated IMRT, and a rectal balloon, with emphasis on long-term biochemical control and treatment-related adverse events in patients with localized prostate cancer.

## Patients and methods

### Patient selection

We performed a retrospective review of patients with localized prostate cancer that were treated with IMRT using the Peacock™ system (Best NOMOS, Pittsburgh, PA, USA) at Houston Methodist Hospital between January 1997 and April 2004. This study was approved by the Institutional Review Board (IRB). Only patients with localized, non-metastatic, and biopsy-proven disease not previously treated were selected for this study. All patients provided informed consent. All biopsies were reviewed at our institution. Patients were staged clinically with a digital rectal examination and pretreatment prostatic-specific antigen (PSA). Computed tomography (CT) scans of the pelvis and bone scans (conventional radiographs or magnetic resonance imaging [MRI] if indicated) were performed for patients at high-risk of metastatic disease.

The patients were stratified using the D’Amico prognostic classification into low—(T1–2a and Gleason score ≤ 6 and PSA ≤ 10 ng/mL), intermediate—(T2b and/or Gleason score = 7 and/or 10 ng/mL < PSA ≤ 20 ng/mL) and high-risk (≥ T2c or Gleason score ≥ 8 or PSA > 20 ng/mL) groups [16].

### Treatment

All patients received IMRT throughout the entire course of radiotherapy. No rectal blocks or field reduction were used. The treatment and planning details were previously described [17]. The unique features of the system are briefly highlighted here. The system delivers radiation using a rotating beam that has its intensity modulated by a special collimator known as the multivane intensity-modulating collimator (MIMiC) (Best NOMOS, Pittsburgh, PA, USA). The MIMiC consists of 40 vanes that are dynamically moved either to allow passage of the beam or to block the beam (a binary system), thereby creating the intensity-modulated pattern. The treatment planning system uses a simulated annealing algorithm that determined the intensity pattern that best achieved the criteria set in the plan.

Patients were immobilized using a Vac-Lok bag and carrier-box system (MEDTEC, Orange City, IA, USA). During simulation, a cystourethrography was performed. A rectal catheter was then inserted, followed by filling the inflatable balloon with 100 cm^3^ of air (E-Z-EM, Westbury, NY, USA). The rectal catheter/balloon was used daily to minimize prostate intrafractional and interfractional movement [18] and provide dosimetric sparing at the rectal wall-balloon surface due to loss of electron equilibrium, as has been previously described [19]. A planning CT scan using 3-mm slice thickness was acquired in the prone position, and images were transferred to the planning workstation for segmentation.

The prostate, seminal vesicles, and critical normal structures (bladder, rectum, and femoral heads) were outlined on each axial image. The entire bladder contents were outlined, while rectal dose-volume constraints were applied to the rectal wall. The planning target volume (PTV) was defined by adding a 0.5 cm margin around the prostate gland and seminal vesicles. A 0.5-cm expansion was chosen as a sufficient margin based on pathologic data examining the radial distance of extraprostatic extension in radical prostatectomy specimens [20]. A PTV dose of 70.00 Gy was prescribed in 35 fractions to the 85% prescription isodose line, resulting in an average mean dose of 76.70 Gy at 2.19 Gy per fraction (normalized total dose at 2 Gy/fraction [NTD_2Gy_] was 80.90 Gy using an α/β ratio of 1.5 Gy). Fractions were delivered on consecutive days, Monday to Friday, over a total of 7 weeks.

### Follow-up

All patients were followed at regular intervals after radiotherapy (every 4 months during the first year, every 6 months in the second to third years, and annually thereafter). Late genitourinary (GU) and gastrointestinal (GI) adverse events were scored according to the Radiation Therapy Oncology Group (RTOG) criteria [21]. Digital rectal examination and PSA detection were performed during follow-up visits. Patients with GU or GI symptoms underwent cystoscopy, sigmoidoscopy, and/or colonoscopy.

### Statistical analysis

The primary endpoint of this study was biochemical relapse-free survival (bRFS), determined by PSA failure defined according to the 2005 American Society for Radiation Oncology (ASTRO) consensus (a rise of at least 2 ng/mL above the PSA nadir). The bRFS was calculated from the date of treatment completion to the date of PSA failure or the date of last follow-up of the patients who were disease-free (these patients were censored) [22, 23]. Univariate and multivariate analyses were performed for bRFS. Examined variables in the univariate analysis included age, Gleason score, T stage, pretreatment PSA, ADT, and D’Amico risk category. In the univariate analysis, the difference in every factor was compared by using the log-rank test. A *P* value of < 0.05 was considered statistically significant. The variables with *P* < 0.05 in univariate analysis were included in multivariate analysis. Multivariate analysis was performed using the Cox proportional hazard regression model. The actuarial probability of Grades 3–4 late adverse events was estimated using the Kaplan–Meier method.

## Results

### Clinicopathologic characteristics of included patients

A total of 596 patients met selection criteria and were included in the final overall analysis. Patient characteristics are summarized in Table 1. Age ranged from 50 to 87 years with a median of 72 years. Metastatic work-up was negative in all patients. None of the patients had any prior definitive treatment for their prostate cancer, such as radical prostatectomy, cryotherapy, and brachytherapy. The median follow-up was 62 months (range 3.7–148 months). Among all patients, 291 (48.8%) were censored.

**Table 1:** Clinicopathologic characteristics of patients with localized prostate cancer who underwent moderately hypofractionated, intensity-modulated radiotherapy

Characteristic	Number of patients [cases (%)]
Gleason score	
≤ 6	274 (46.0)
7	240 (40.3)
≥ 8	82 (13.8)
T stage	
T1–T2b	577 (96.8)
T2c	5 (0.8)
≥ T3	14 (2.3)
Pretreatment PSA level (mg/dL)	
≤ 10	467 (78.4)
> 10 and ≤ 20	98 (16.4)
> 20	31 (5.2)
D’Amico category	
Low risk	226 (37.9)
Intermediate risk	264 (44.3)
High risk	106 (17.8)
ADT in risk groups	
Low-risk	48 (21.2)
Intermediate-risk	208 (78.8)
High-risk	105 (99.1)

*PSA* prostate-specific antigen, *ADT* androgen deprivation therapy

### bRFS and outcome according to risk group

**Fig. 1:**
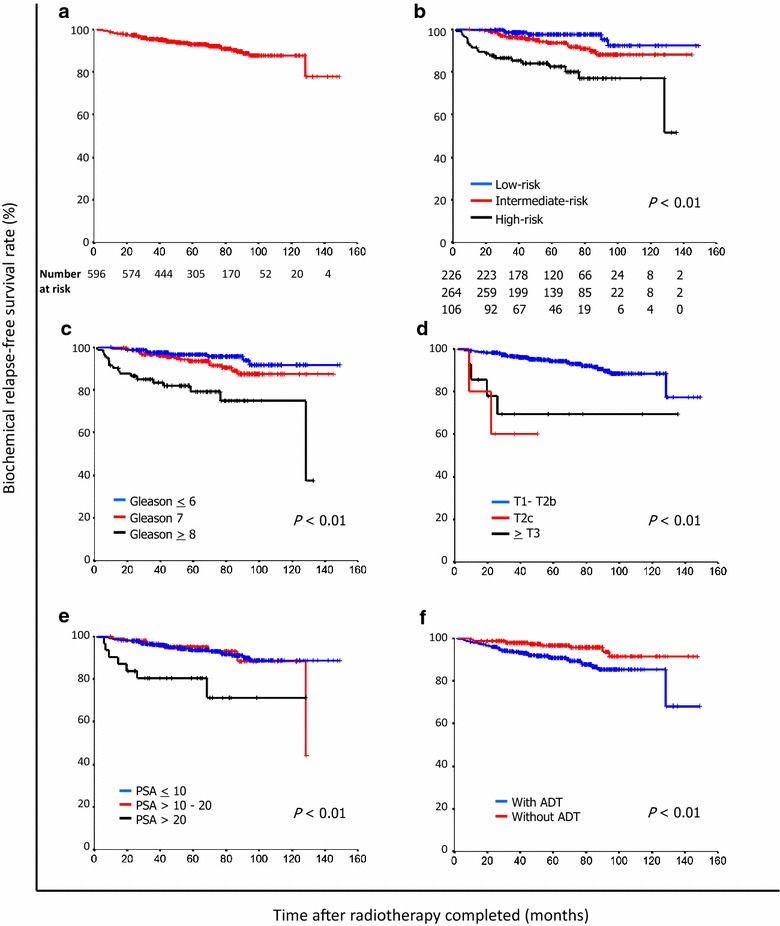
Kaplan–Meier curves of biochemical relapse-free survival (bRFS) of patients with localized prostate cancer who underwent moderately hypofractionated, intensity-modulated radiotherapy. **a** bRFS of the entire cohort. **b** bRFS stratified by D’Amico risk group. **c** bRFS stratified by Gleason score. **d** bRFS stratified by T stage. **e** bRFS stratified by pretreatment PSA. **f** bRFS stratified by the use of androgen deprivation therapy (ADT)

For the entire cohort, the 5- and 10-year bRFS rates were 92.7% and 87.7% (Fig. 1a). In univariate analysis, lower D’Amico risk, lower Gleason score, less advanced T stage, lower pretreatment PSA, and without ADT were significant predictors of prolonged bRFS (all *P* < 0.01) (Table 2 and Fig. 1b–f). Gleason score and T stage, but not D’Amico risk group, emerged as significant predictors of bRFS in multivariate analysis (Table 3).

**Table 2:** Univariate analysis for biochemical relapse-free survival (bRFS) of patients with localized prostate cancer

Variate	5-year bRFS rate (%)	*P* value
Age (years)		
≤ 72	91.8	0.57
> 72	93.6	
Gleason score		
≤ 6	96.7	< 0.01
7	93.5	
≥ 8	79.3	
T stage		
T1–T2b	94.1	< 0.01
T2c	60.0	
≥ T3	69.3	
Pretreatment PSA level (mg/dL)		
≤ 10	93.5	< 0.01
> 10 and ≤ 20	95.2	
> 20	80.2	
D’Amico risk category		
Low risk	96.9	< 0.01
Intermediate risk	93.3	
High risk	82.0	
ADT		
No	96.6	< 0.01
Yes	90.7

**Table 3:** Cox multivariate analysis for bRFS of patients with localized prostate cancer

Covariate	b	SE	Wald	*P* value	Exp(b)
Gleason score	0.88	0.21	17.7	< 0.01	2.42 (1.60–3.66)
T stage	0.76	0.24	9.6	< 0.01	2.13 (1.32–3.44)
Pretreatment PSA level (mg/dL)	0.34	0.25	1.8	0.18	1.40 (0.85–2.31)
D’Amico risk category	0.05	0.51	0.01	0.92	1.05 (0.39–2.86)
ADT	− 0.03	0.46	0.01	0.95	0.97 (0.39–2.40)

*b* regression coefficient b, *SE* stand error of regression coefficient b, *Wald* Wald statistic (b/SE)^2^, *Exp(b)* exponentiation of the b coefficient

### Patterns of failure

Forty-six patients (7.7%) experienced biochemical failure: 32 (5.4%) had only biochemical evidence without clinically/radiographically detectable local or distant recurrence, 6 (1.0%) had biochemically detected and clinically/radiographically detectable, biopsy-proven local failure and 8 (1.3%) had biochemical and radiographic evidence of distant failure. Biochemical failure rates were 3.1%, 7.2%, and 18.9% for the low-, intermediate-, and high-risk groups, respectively. Seven of 8 cases of distant failure were observed in the high-risk group, and no distant failure was seen in the low-risk group.

### Late adverse events

Limited late adverse events were observed in our study population, as evidenced by 5-year actuarial rates of 8.6% (51 patients) for ≥ Grade 2 GI adverse events and 19.5% (116 patients) for ≥ Grade 2 GU adverse events. However, several patients did experience severe late morbidity. Five patients (0.8%) developed Grade 3 rectal adverse events, typically rectal bleeding requiring cauterization. One patient (0.2%) experienced a Grade 4 fistula and required colostomy 26 months after radiotherapy. Six patients (1.0%) developed Grade 3 GU adverse events, typically manifesting as frequent hematuria. No Grade 4 GU adverse events were observed.

## Discussion

We have presented the long-term outcomes of a large cohort of patients with IMRT-treated prostate cancer. Study strengths include mature follow-up and homogeneous treatment techniques, as all patients underwent full-course IMRT and were treated in the prone position with a rectal balloon using a uniform, moderate hypofractionation regimen, and using consistent treatment planning and delivery methods at a single facility. The results demonstrate good disease control and acceptable rates of adverse events.

We opted to use an endorectal balloon for our patients due to several advantages. The balloon provides a constant rectal filling that immobilizes the prostate by pushing it towards the pubic bone. In addition, the balloon pushes the dorsal wall of the rectum away from the prostate, resulting in improved rectal sparing and reduced adverse events. Our group has previously published on the reduced dose to the rectum at the air-tissue interface between the balloon and the rectal wall [19]. However, the rectal balloon pushes the anterior wall of the rectum closer to the prostate; therefore, there is concern that because part of the rectum is close to the high dose region, there might actually be an increase in rectal adverse events.

The low α/β ratio estimates for prostate cancer have generated substantial interest in higher-than-conventional fraction sizes [24]. As a result, multiple regimens have been clinically implemented, with fraction doses ranging from approximately 2.2–10 Gy. However, given the excellent therapeutic outcomes, both in terms of disease control and rates of adverse events, achievable with modern, dose-escalated radiotherapy for prostate cancer using image-guided irradiation and conventional fractionation, the adoption of alternative fractionation regimens necessitates careful clinical validation prior to widespread implementation. Extreme hypofractionation using fraction doses ≥ 6 Gy typically requires stereotactic techniques [25] and is outside the scope of the present study. Nevertheless, our dataset provides an opportunity for outcomes comparison with other moderate hypofractionation regimens employing fraction doses of approximately 2.2–4 Gy. To facilitate comparison between different fractionation regimens discussed in this section, doses are represented as NTD_2Gy_ using the Fowler formalism [26, 27] with an α/β ratio of 1.5 Gy (Table 4).

**Table 4:** Summary of studies on prostate cancer treated with radiotherapy using hypofractionation

Author and publication, year	Technique	No. of patients	Stage T1–2 (%)	ADT (%)	Median follow-up (months)	Total dose (Gy)	Dose per fraction (Gy)	NTD_2Gy_ (Gy_2_)	5-year bRFS rate (%)	Rate of late GI events (%)		Rate of late GU events (%)	
										Grade ≥ 2	Grade ≥ 3	Grade ≥ 2	Grade ≥ 3
Kupelian et al. [12], 2007	IMRT	770	95	60	45	70	2.5	80.0	83	4.5	1.4	5.3	0.1
Pollack et al. [13], 2013	IMRT	151	86	45	68.4	70.2	2.7	84.4	76.7	18.1	2	21.5	4
Arcangeli et al. [14], 2012	3D-CRT	83	65	100	70	62	3.1	81.5	85	17	1.2	16	1.2
Kuban et al. [15], 2010	IMRT	102	NR	21	55.2	72	2.4	80.2	96	11	3	19	0
Lukka et al. [28], 2005	3D-CRT	466	100	0	68.4	52.5	2.6	61.5	40	NR	1.3	NR	1.9
Yeoh et al. [29], 2011	2D, 3D-CRT	108	100	0	90	55	2.75	66.8	55.9	NR	NR	NR	NR
Leborgne et al. [30], 2011	3D-CRT	114	95.6	NR	49	60 63	3 or 3.15	77.1 or 83.7	89	4.4	0.9	4.4	1.8
Norkus et al. [31], 2009	3D-CRT	47	98	0	12	57	3 (13 fraction) or 4.5 (4 fraction)	81.0	NA	NR	NR	NR	NR
Dearnaley et al. [32], 2016	IMRT	1074	91	97	62.4	60	3	77.1	90.6	11.9	< 1	11.7	< 1
		1077	90	97		57		73.3	85.9	11.3	< 1	6.6	1
Incrocci et al. [33, 34], 2016	IMRT	407	48	66	60	64.6	3.4	90.4	80.5	21.9	3.3	41.3	19.0
Lee et al. [35], 2016	3D-CRT or IMRT	550	100	0	69.6	70	2.5	80.0	86.3	22.4	4.1	29.7	3.5
*Present series*	*IMRT*	*596*	*98*	*61*	*62*	*76.70*	*2.19*	*80.9*	*92.7*	*8.5*	*1.2*	*19.4*	*1.1*

*bRFS* biochemical relapse-free survival, *ADT* androgen deprivation therapy, *NTD*_*2Gy*_ normalized total doses in 2 Gy/fraction equivalents with an α/β ratio of 1.5 Gy, *GI* gastrointestinal, *GU* genitourinary, *3D-CRT* 3-dimensional conformal radiotherapy, *NR* not reported, *NA* not analyzed, *IMRT* intensity-modulated radiotherapy, *EQD*_*2*_ equivalent dose in 2 Gy/fraction

Early hypofractionation randomized trials used 3D-CRT technique with relatively low total doses ranging from 52.5 to 55 Gy in 20 fractions (NTD_2Gy_, 61.5–66.8 Gy_2_) [28, 29]. As expected, treatment outcomes were suboptimal, with 5-years bRFS ranging from 40% to 55.9%. Other 3D-CRT series, however, employed more aggressive fractionation regimens, with NTD_2Gy_ values ranging from 77.1 to 83.7 Gy_2_ and have reported improved outcomes (5-year bRFS rates generally above 80%, with some exceptions) despite including patients with high-risk disease [14, 30–32].

A phase I/II trial by Kupelian et al. [12] was a large prospective study of hypofractionated IMRT and prescribed 70 Gy in 28 fractions (NTD_2Gy_ = 80 Gy_2_). At a median follow-up of 45 months, the authors reported 5-year bRFS rate of 83% for the entire cohort, and 94%, 83%, and 72% for low-, intermediate-, and high-risk cohorts, respectively. Severe (Grade ≥ 3) late rectal and urinary adverse events were uncommon. Results from two additional randomized trials have been recently reported. Pollack et al. [13] tested a hypofractionation regimen delivering 70.2 Gy in 26 fractions (NTD_2Gy_ = 84.4 Gy_2_). Among 151 patients and with a median follow-up of 68.4 months, the observed 5-year bRFS rate was 76.7%. The 5-year rates of Grade ≥ 2 GI and GU adverse events were 18.1% and 21.5%. Similarly, Kuban et al. [15] employed 72 Gy in 30 fractions (NTD_2Gy_ = 80.2 Gy_2_) and reported, at a median follow-up of 4.6 years, a 5-year bRFS rate of 96% for the 102 patients in the hypofractionation group. There were 9 patients with Grade 2 and 2 with Grade 3 GI adverse events, for 5-year actuarial rates of 11% and 3%. Overall, the 5-year rate of Grade ≥ 2 GU adverse events 19%.

More recently, three randomized, phase III trials published mature results comparing conventionally fractionated radiotherapy with hypofractionated radiotherapy. The largest was CHHiP [32], which was a non-inferiority study that randomized 3216 patients with localized prostate cancer to conventional (74 Gy delivered in 37 fractions) or one of two hypofractionation regimens (60 Gy in 20 fractions or 57 Gy in 19 fractions, NTD_2Gy_ = 77.1 or 73.3 Gy_2_, respectively) delivered via intensity-modulated techniques. The observed 5-year bRFS rate was 88.3% in the 74 Gy group vs. 90.6% and 85.9% in the hypofractionation groups. The study found that 60 Gy was non-inferior to 74 Gy in terms of bRFS, but non-inferiority could not be claimed for 57 Gy compared with 74 Gy. The rates of Grade ≥ 2 GI and GU adverse events were 13.7% and 9.1% in the 74 Gy group, 11.9% and 11.7% in the 60 Gy group, and 11.3% and 6.6% in the 57 Gy group, respectively. The authors recommended hypofractionated radiotherapy as a new standard of care for localized prostate cancer.

Another large randomized, phase III trial [33, 34] was conducted at 7 Dutch centers. It enrolled 804 patients with localized prostate cancer and randomized them to either hypofractionated radiotherapy of 64.6 Gy (19 fractions of 3.4 Gy, 3 fractions per week, NTD_2Gy_ = 90.4 Gy_2_) or conventionally fractionated radiotherapy of 78 Gy (39 fractions of 2 Gy, 5 fractions per week). The 5-year bRFS rate was 80.5% for patients in the hypofractionation group and 77.1% for those in the conventional fractionation group. The 3-year rate of Grade ≥ 2 GU adverse events was 39.0% in the conventional fractionation group and 41.3% in the hypofractionation group. In addition, there was a significant increase in the 3-year rate of Grade ≥ 3 GU adverse events in the hypofractionation group compared with that in the conventional fractionation group (19% vs. 12.9%). The 3-year rate of Grade ≥ 2 GI adverse events was 17.7% in the conventional fractionation group and 21.9% in the hypofractionation group. The authors concluded that their hypofractionation regimen could not be regarded as the new standard of care.

The third trial with recent results was conducted by NRG [National Surgical Adjuvant Breast and Bowel Project (NSABP), the Radiation Therapy Oncology Group (RTOG), and the Gynecologic Oncology Group (GOG)] Oncology (0415) [35], which randomized 1092 men with low-risk prostate cancer to hypofractionated radiotherapy (70 Gy in 28 fractions, NTD_2Gy_ = 80 Gy_2_) versus conventionally fractionated radiotherapy (73.8 Gy in 41 fractions, NTD_2Gy_ = 69.6 Gy_2_). 3D-CRT or IMRT were allowed. The estimated 5-year bRFS rate was 85.3% in the conventional fractionation group and 86.3% in the hypofractionation group. There was an increase in late Grade ≥ 2 GI and GU adverse events in the hypofractionation group (22.4% and 29.7%, respectively) compared with the conventional fractionation group (11.4% and 20.5%). The difference in the rate of late Grade 2 GI adverse events reached statistical significance. The authors concluded that hypofractionation was non-inferior to conventional fractionation.

These recently published results give credence to the idea that hypofractionated radiotherapy is an effective treatment for prostate cancer in terms of disease control. There is concern, however, over the possibility of increased late GI and GU adverse events as compared with conventional fractionation, although overall rates remain low. This concern, along with the heterogeneity in the various hypofractionation regimens, has led some clinicians to give pause before offering hypofractionated radiotherapy as standard of care therapy. However, the recently published data likely does obligate practitioners to discuss the risks and benefits of hypofractionated radiotherapy with their patients, particularly those who are interested in a shortened course of radiotherapy.

At its inception, our program sought to safely improve biochemical control rates by delivering an increased biological effective dose to the prostate target volume while maintaining a constant number of fractions relative to the conventional doses of 66–70 Gy which predated the modern dose escalation era. The long-term treatment outcomes using a mean dose prescription of 76.7 Gy in 35 fractions (NTD_2Gy_ = 80.9 Gy_2_) demonstrate the safety and efficacy of this fractionation regimen by virtue of excellent long-term bRFS and low rates of severe late GI and GU adverse events, which falls in line with the recently published randomized data.

We recognize that our study has important limitations, including its single-institution, single-arm, retrospective design, lack of detailed dose-volume histogram analysis, omission of image-guided delivery techniques, and potential bias in the use of hormonal therapy, particularly among low-risk patients. Nevertheless, the more recent published studies reported similar outcomes and give some support to our data.

## Conclusions

Long-term bRFS and adverse event outcomes in patients with localized prostate cancer treated using a moderately hypofractionated IMRT regimen are encouraging. Based on this large single-institution study, in combination with the recently published data from three randomized clinical trials, moderate hypofractionation represents a safe, efficacious regimen in the treatment of localized prostate cancer that should be discussed with patients interested in a shortened course of treatment.

## Abbreviations

bRFS: biochemical relapse-free survival
3D-CRT: three-dimensional conformal radiotherapy
IMRT: intensity-modulated radiotherapy
ADT: androgen deprivation therapy
NCCN: National Comprehensive Cancer Network
PSA: prostatic specific antigen
MRI: magnetic resonance imaging
GS: Gleason score
MIMiC: multivane intensity modulating collimator
PTV: planning target volume
GU: genitourinary
GI: gastrointestinal
